# Three-Dimensional Ordering of Nematic Liquid Crystals with Azimuth and Tilt Controlled by Patterned Photoalignment and Selective Polymer Stabilization

**DOI:** 10.3390/polym17030418

**Published:** 2025-02-05

**Authors:** Marta Kajkowska, Miłosz Sławomir Chychłowski, Michał Ptaszek, Sławomir Ertman

**Affiliations:** Faculty of Physics, Warsaw University of Technology, Koszykowa 75, 00-662 Warsaw, Poland; milosz.chychlowski@pw.edu.pl (M.S.C.); michal.ptaszek.stud@pw.edu.pl (M.P.)

**Keywords:** liquid crystal, photoalignment, polymer stabilization, polymer-stabilized liquid crystal

## Abstract

In this paper, we present a novel approach for advanced, three-dimensional patterned ordering of nematic liquid crystals. Our method allows for simultaneous control of azimuth and tilt of molecules by using a two-step process based on patterned photoalignment (used to define azimuth) followed by selective polymer stabilization of molecules reorientated with an electric field (used to define tilt). We demonstrate that those two subsequent processes, realized with high-resolution patterned illumination with UV light, allow us to obtain multiple microdomains with independently controlled tilt and azimuth. It opens possibilities to create complex three-dimensional distributions of director within a single liquid crystal cell, which is impossible with any other technique so far. Moreover, although the polymer-stabilization process is used, it is still possible to retune the tilt of the molecules; however, the electric field intensity needed for tuning is slightly higher than in the non-polymerized areas of the sample.

## 1. Introduction

Liquid crystal-based composite materials have been widely studied over the last few decades [1,2,3]. Materials consisting of a liquid crystal (LC) base and polymer dopant in low concentration (<10%), also known as polymer-stabilized LCs, are of particular interest. This is due to the combination of unique electro-optical properties of LCs with added stabilization of LC orientation [4,5,6] and improved thermal stability [7,8] of the material thanks to the presence of a polymer network formed between the LC molecules. Polymer stabilization of LCs has been demonstrated to allow for control of LC ordering by stabilization of any desired ordering of LC molecules [9,10], which in the case of planarly oriented nematics results in increased threshold voltage [4]. Selective photopolymerization technique has been successfully implemented for the fabrication of optical elements such as diffractive and refractive LC lenses [6,11,12], diffraction gratings (including ones with polarization-dependent period value) [10,13] as well as waveguides [14,15,16,17]. Aside from that, polymer stabilization was also utilized for the stabilization of self-organized structures in liquid crystals, such as a diffraction grating with sub-micrometer periodicity [18] or one-dimensional photonic crystals [5]. An interesting result was also presented by Yu-Cheng Hsiao and Wei Lee, who utilized photopolymerization of grid-patterned multidomain chiral LC to ensure a wide range of viewing angles (from −60° up to 60°) [19]. Other noteworthy results were obtained by Runwei Yu et al., who used a cholesteric LC as a multicolor base in which the selection of refracted color was controlled by irradiation time with a UV light source [20].

Enforcing a specific LC orientation is often achieved via photoalignment [21,22,23,24,25,26,27]. This alignment method utilizes photosensitive alignment layers to control LC azimuth in the LC cell. Its most significant advantage over aligning the LC via rubbing the polymer layers is the possibility of obtaining variable LC azimuth in the LC cell. Similar to selective polymer stabilization, patterned photoalignment has been used to obtain structures with complex arrangements of LC molecules. This technique was proven useful for the fabrication of diffractive optical elements and displays [28,29,30,31,32,33,34,35]. Some groups demonstrated quasi-3D control of LC ordering by using patterned photoalignment either to achieve anchoring on the surface of LC cells filled with chiral LCs [36,37] or through irradiation of orthogonal patterns on the top and bottom of the LC cell [38,39,40].

The research presented in this paper was aimed at the development of a method allowing for full three-dimensional control of nematic LC’s ordering. To achieve this, we utilized the LC cell with an azo dye layer to perform patterned photoalignment to control LC azimuth and infiltrated it with nematic LC-based composite, which was then selectively polymerized in the external electric field to stabilize the tilt of LC molecules. The obtained results prove that it is possible to achieve an LC arrangement that is a superposition of the azimuth enforced by the azo dye alignment layer and tilt stabilized with the polymer network. The proposed LC ordering technique is a significant improvement compared to the ones already described in the literature, as it allows independent microdomains with any designed tilt and azimuth of the LC to be created. It can be implemented to fabricate complex LC-based structures for various photonic applications.

## 2. Materials and Methods

### 2.1. Composite and LC Cells

The composite LC material was based on a nematic LC mixture E7 doped with 5 wt% of a 9:1 mixture consisting of RM257 monomer (97%, SYNTHON Chemicals, Bitterfeld-Wolfen, Germany, CAS:174063-87-7) and 2,2-dimethoxy-2-phenylacetophenone photoinitiator (99%, Sigma Aldrich, St. Louis, MO, USA, CAS: 24650-42-8) [41], respectively. The material was fabricated by mixing monomer and photoinitiator in desired proportions and adding the LC afterward. This part of the preparation procedure was conducted at room temperature, and the light source in the room did not contain wavelengths within the absorption spectrum of the photoinitiator. Uniform distribution of the compounds was obtained by mixing the material in an ultrasonic bath at a temperature above LC’s clearing point for 30 min. The finished material was stored in a UV-impermeable container to avoid undesired polymerization. An in-depth analysis of the morphology of LC-based composites consisting of the same materials as those used in this research was done by Che-Ju Hsu et al. [42]. The authors described differences in polymer network density for various concentrations of RM257 in the composite as well as polymer network density’s dependence on the time and intensity of UV irradiation.

Two types of empty LC cells have been used at various stages of this research. Tests of photoalignment and experiments with two-step illumination (photoalignment and photopolymerization) have been performed in self-made, 12 µm thick LC cells based on ITO-coated glass spin-coated with SD1 azo dye [43], which was used as a photo aligning material. The ITO-coated glass was purchased from Ossila, and its thickness was 1.1 mm. In the case of tests aimed at optimizing photopolymerization in an external electric field, commercially available LC cells (purchased from the Military University of Technology) with ‘classic’ planar alignment layers (alignment obtained via unidirectional rubbing) have been used. These cells also had 12 µm spacing and ITO coating; however, the glass thickness was 0.5 mm.

### 2.2. Two-Step Process Based on Patterned Photoalignment Followed by Selective Polymer Stabilization

**Figure 1:**
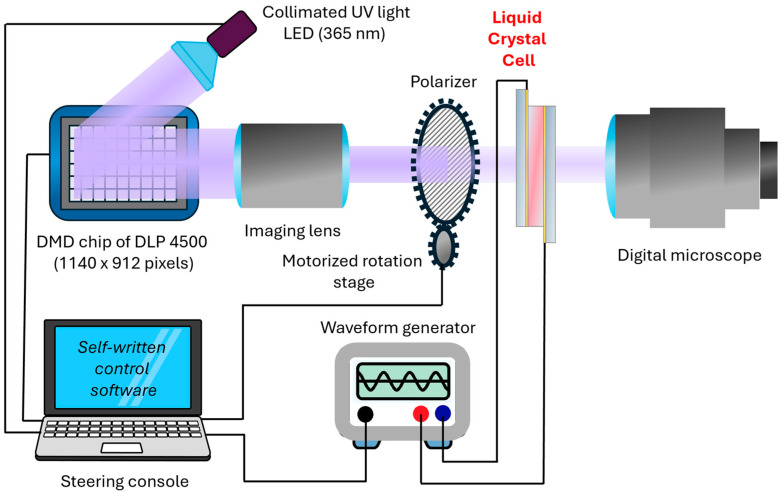
Scheme of the setup used for both photoalignment and photopolymerization. The polarizer is removed from the setup for photopolymerization.

The sample fabrication procedure was divided into two stages: photoalignment and photopolymerization. In both stages, illumination with micro-patterned UV light was required, and it was realized in the setup, which is schematically presented in Figure 1. The main part of the setup is a projection system based on DLP LightCrafter 4500 (Texas Instruments (Dallas, TX, USA)), in which a digital micromirror device (DMD) chip was illuminated with a 365 nm UV LED (a modified light engine has been supplied by EKB Technologies (Bat Yam, Israel)). The DMD chip consists of more than one million micro-mirrors (7.6 × 7.6 μm), arranged in 912 columns by 1140 rows with diamond pixel array geometry. To control the resolution of illumination, we used an optical imaging lens to rescale the size of the pixel—it was possible to scale the pixel size in the range from about 3 × 3 μm to 10 × 10 μm. LC cells have been positioned precisely to ensure that the image plane of the projection setup is exactly in the center of the cell. In the photoalignment step, polarized light was used, and the azimuth of the polarizer was controlled with dedicated software (it was synchronized with the subsequent image pattern displayed by the DLP device). A similar setup has recently been used for patterned alignment of LC molecules in microcapillaries [44] and selective polymer stabilization of an LC-based composite in LC cells [10]. In the photopolymerization step, the LC tilt was steered with a waveform generator, controlled with custom software, and synchronized with displayed images. The whole process was visually controlled with a digital microscope placed at the back of the LC cell, which was also used for the proper positioning of the sample.

During photoalignment, an empty LC cell was positioned in the image plane of the setup and irradiated with ~2 mW/cm^2^ intensity for 10 min per polarization azimuth. This stage of the irradiation procedure required the use of a polarizer in the setup, as linearly polarized light with controlled azimuth was needed to irradiate the alignment layer properly. The SD1 azo dye used for photoalignment provides planar orientation of LC with azimuth defined by the polarization direction of UV light (in the case of the SD1 material, the molecules of nematic LC align perpendicularly to the azimuth of linearly polarized UV light).

**Figure 2:**
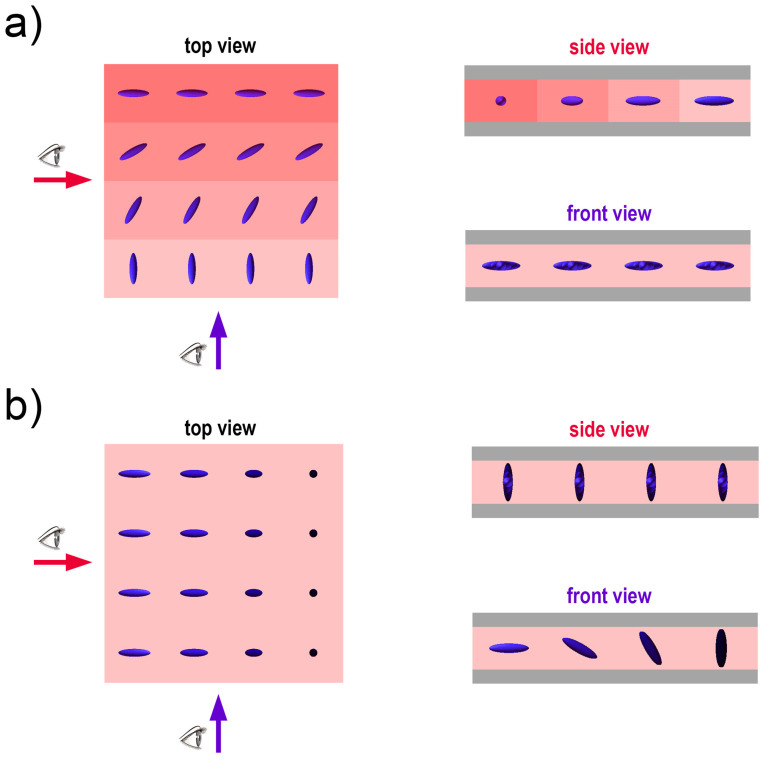

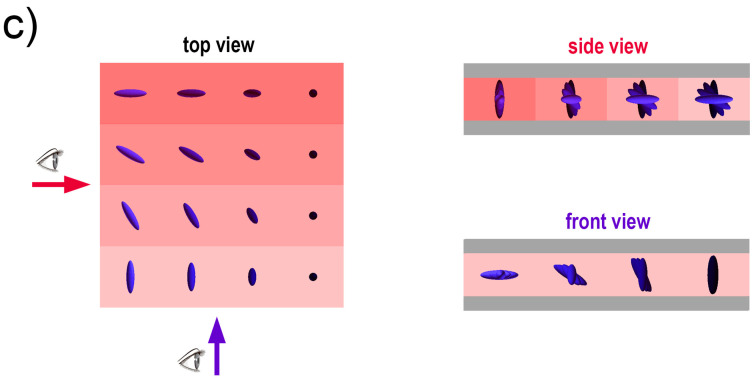
Schematic representation of LC ordering obtainable via: (**a**) patterned photoalignment with linearly polarized light to obtain planar alignment with variable azimuth; (**b**) selective photopolymerization of LC molecules reoriented with an external electric field to control the tilt of the molecules; (**c**) combination of azimuth control with photoalignment and tilt control with polymer stabilization for 3D control of LC arrangement.

After irradiation of the alignment layer, the LC-based composite was introduced into the LC cell using capillary forces. The irradiation intensity was reduced to ~0.7 mW/cm^2^ to avoid heating of the LC due to absorption of radiation. For this irradiation stage, the polarizer was removed from the setup as the polarization of light has no influence on the photopolymerization process, but further use of polarized light could possibly disrupt the previously obtained alignment [34]. The sample was repositioned to account for the change in the optical path resulting from the removal of the polarizer. For the purpose of LC control during polymerization, the LC cell was connected to a function generator to set the desired tilt of LC molecules with an external AC electric field (1 kHz square waveform). The irradiation time per LC orientation was dependent on the LC cell used in the experiment—for the preliminary tests, it was 10 min; however, the final samples fabricated in the self-made cells required 30 min of irradiation due to the use of thicker glass and UV absorption by the azo dye. Figure 2 shows the general idea of LC control with photoalignment (Figure 2a), photopolymerization (Figure 2b), and a combination of both techniques for 3D control (azimuth and tilt) of the molecules (Figure 2c).

### 2.3. Sample Analysis

The samples were analyzed under a digital microscope (Keyence VHX-5000 (Keyence Corporation, Osaka, Japan)) by placing each sample between crossed polarizers to observe differences in phase delay introduced by the LC and thus determine its molecular arrangement. The photoaligned samples were examined via rotation between the polarizers as the change in LC orientation was in the plane of the LC cell. In the case of photopolymerization, the LC orientation was changing in the plane perpendicular to the LC cell’s surface. For this reason, stabilization of the desired LC tilt was verified by applying an AC electric field to the sample and comparing the appearance of polymerized and non-polymerized LC for the voltage values used during polymerization.

## 3. Results

The preliminary tests were aimed at establishing optimal irradiation parameters for photoalignment and photopolymerization before the two techniques were combined to obtain 3D control of LC arrangement.

### 3.1. Photoalignment

**Figure 3:**
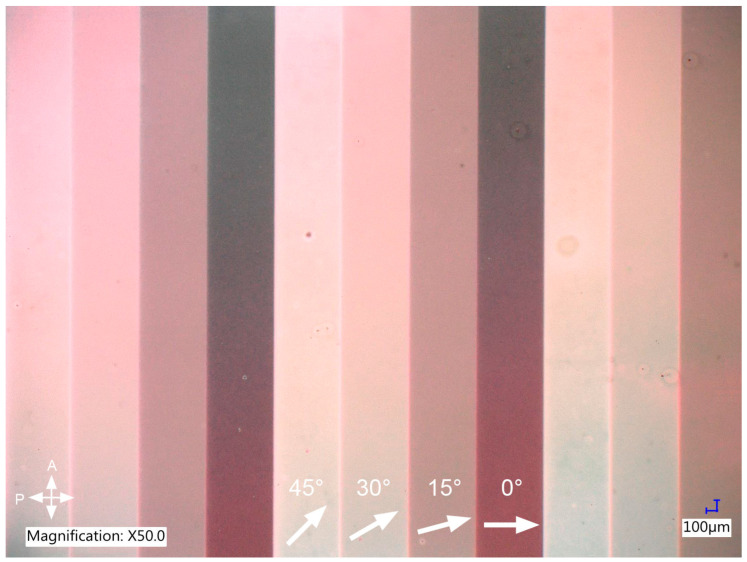
Control of azimuth of planarly oriented LC obtained for 10 min of selective UV irradiation per polarization direction. The sample was prepared in a self-made, 12 µm LC cell and filled with LC-based composite after irradiation. The LC director in each type of stripe of the periodic stripe pattern is marked with an arrow, and its angle with respect to the polarizer is given above it.

The first stage of preliminary research focused on determining both the necessary intensity and time of simultaneous irradiation of the top and bottom alignment layers and an optimal method of infiltrating the LC cell after irradiation. The goal was to obtain a pattern with variable LC azimuth and sharp borders between areas with different LC arrangements. The LC cell was selectively irradiated in each test with four different polarization directions in the range from 0 to 45 degrees with respect to the cell’s edge. Such values were chosen to allow the best visualization of varying LC azimuth under a polarizing microscope. Optimization of the irradiation procedure was performed for undoped E7 to eliminate the possible influence of the dopant on LC orientation. The infiltration of a photoaligned cell was done with LC in the isotropic phase to ensure the most uniform LC orientation possible. Next, the fabrication procedure with optimal irradiation parameters was repeated, but the cell was filled with an E7-based composite this time. It turned out that infiltration with the composite in the isotropic phase was problematic as the monomer separated from the LC and agglomerated in random spots, which was most likely due to the temperature difference between the LC material and the cell. Consequently, the cell was filled with the composite in the nematic phase, and the results can be seen in Figure 3.

It can be seen that introducing the composite in the nematic phase did not cause any visible defects in the LC arrangement, and the distribution of monomer dopant seems uniform. As expected, photoalignment allowed us to obtain domains with planar molecular alignment and various azimuths. The difference in LC azimuth results in different brightness of each stripe observed under a polarizing microscope. The dark region is observed when the azimuth of planar orientation is parallel or perpendicular to the transmitting axis of a polarizer. On the other hand, the brightest stripe is observed when the angle between the LC azimuth and the polarizer’s transmitting axis is equal to 45 degrees, as it results in the largest phase shift between two orthogonal polarization components. Of course, if the sample would be rotated axially, the brightness of each stripe would gradually change. By using this simple method, we were able to verify that LC molecules in each subdomain have planar alignment with the expected azimuth.

### 3.2. Photopolymerization

**Figure 4:**
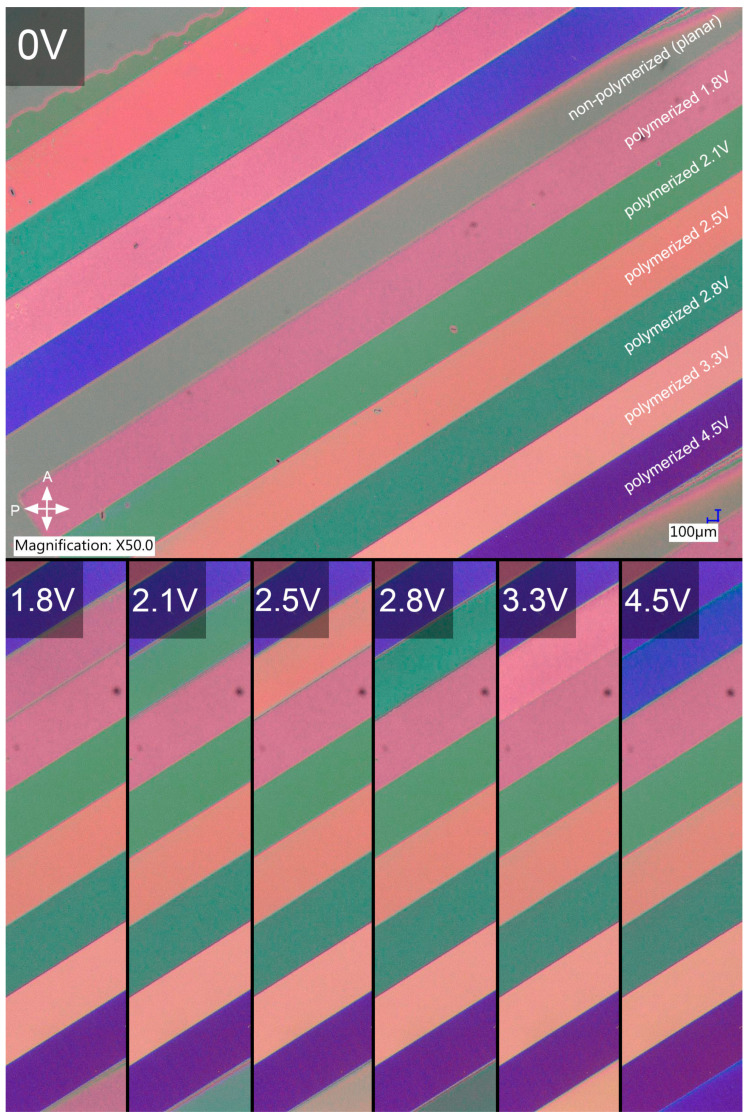
Polymer stabilization of LC tilt obtained via the application of an external electric field and selective UV irradiation for 10 min per voltage value. The sample was prepared in a commercially available 12 µm LC cell with planar alignment layers. The top image shows the polymerized sample with no external electric field applied to it and the AC voltage values used during the photopolymerization of each stripe (one stripe in the periodic stripe pattern was left non-polymerized to be used as a reference). The bottom images show a comparison of LC orientation between the reference stripe and the polymerized ones for each voltage value used during polymerization. Slight color variation between the sets of stripes is caused by a minor tilt of the LC cell under the microscope.

The focus of further preliminary research was on obtaining stabilization of the LC tilt set in an external electric field. The sample was selectively irradiated for different voltage values to get different tilts of the molecules, and one stripe in the periodic pattern was left non-polymerized to be used as a control region. The tests were performed in commercially available LC cells with planar alignment layers to eliminate any possible influence of the azo dye on photopolymerization efficacy and quality. The irradiation intensity for this part of the research was lowered compared to photoalignment to avoid heating the LC composite due to radiation absorption. The sample obtained for optimized process parameters is presented in Figure 4.

Polymer stabilization of LC tilt was not only successful in the sense that the molecular arrangement obtained in the external AC electric field was preserved after photopolymerization. Figure 4 clearly shows that the effective birefringence of the composite for each voltage value remained identical after irradiation, which is evident in the images presented in the bottom row, where the polymerized stripes are compared with the control one. For each voltage amplitude used during irradiation, the color of a stripe polymerized for this specific voltage is identical to the non-polymerized one when an identical voltage value is applied to the sample. Moreover, no visible light scattering on the polymer network was introduced.

### 3.3. Photoalignment and Photopolymerization

**Figure 5:**
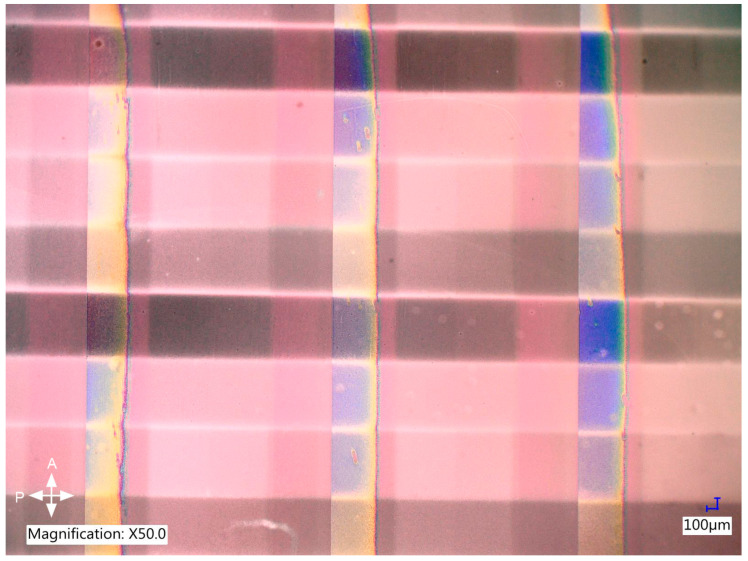
Control of azimuth (horizontal stripes) and tilt (vertical stripes) of LC molecules obtained by photoalignment and subsequent photopolymerization. The sample was prepared in a self-made, 12 µm LC cell by UV irradiation of the alignment layer, filling the cell with LC composite after irradiation, and photopolymerizing the composite. In each process, the irradiation time per polarization direction/voltage value was 10 min. It can be seen that polymer stabilization of LC tilt was not successful.

The first attempt to obtain 3D control of LC orientation utilized the process parameters determined in the preliminary research: 10 min of irradiation per azimuth/tilt with ~2 mW/cm^2^ and ~0.7 mW/cm^2^ irradiation intensity for photoalignment and photopolymerization, respectively. The results of this procedure can be seen in Figure 5.

**Figure 6:**
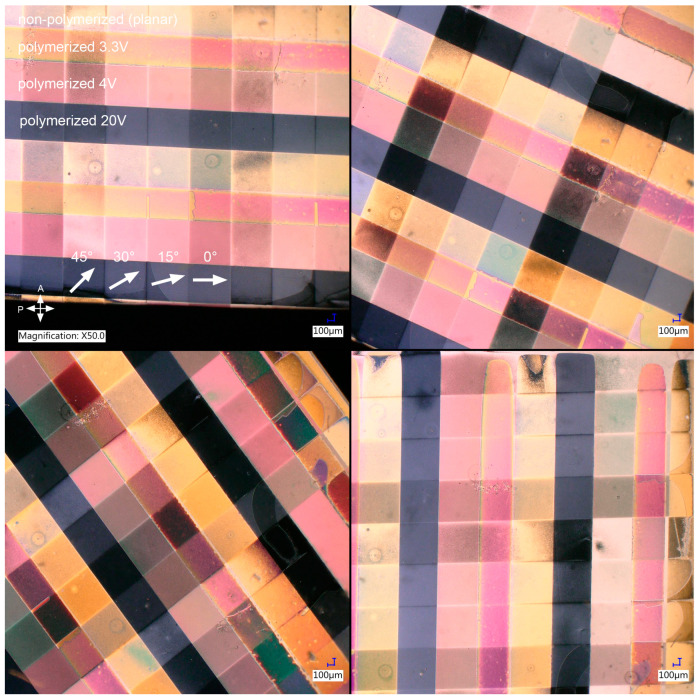
Control of azimuth (vertical stripes in top left picture) and tilt (horizontal stripes in top left picture) of LC molecules obtained by photoalignment and subsequent photopolymerization. The sample was prepared in a self-made, 12 µm LC cell by UV irradiation of the alignment layer, filling the cell with LC composite after irradiation and photopolymerization of the composite. The irradiation time per polarization direction was 10 min, and per voltage value it was extended to 30 min to account for the increased thickness of the LC cell compared to Figure 4. The sample is observed without an electric field applied to the LC cell and rotated between crossed polarizers to demonstrate 3D control of LC ordering. The top left picture shows the LC director in each stripe in the periodic stripe pattern marked with an arrow and its angle with respect to the polarizer, as well as the voltage values used during photopolymerization.

It is clear that photoalignment with the parameters established during the preliminary research was successful; however, polymer stabilization was not, despite multiple attempts. The stripes with different LC tilts were somewhat visible, but it appeared that the tilt was not stabilized properly. Based on the analysis of the samples’ electro-optical behavior under a microscope, it appeared that this was due to the incomplete formation of polymer chains. It was concluded that a result so vastly different from Figure 4 was probably due to a change of the LC cell in which polymerization was conducted—the cell used for preliminary tests was made of significantly thinner glass and did not have an azo dye layer. The increase in glass thickness, along with the introduction of azo dye, which also absorbs UV, must have caused a decrease in the intensity of UV light reaching the LC-based composite due to the absorption of radiation. The irradiation time of photopolymerization was gradually increased to counteract this effect until the proper value was determined. Successful fabrication of a sample required 30 min of irradiation per LC tilt with the same intensity as in preliminary tests. The final sample, shown in Figure 6, was created by photoalignment (10 min of irradiation per azimuth with ~2 mW/cm^2^), filling the cell with the composite in the nematic phase, and consequent polymer stabilization of LC tilt by 30 min of irradiation with ~0.7 mW/cm^2^ per voltage value.

It is evident that modification of polymerization parameters allowed proper stabilization of the LC tilt for each voltage value. Analysis of the sample under a polarizing microscope clearly demonstrates that full 3D control of LC orientation was achieved. The color and brightness of each region, being a superposition of orthogonal stripes with different azimuth and tilt, changes differently when the sample is rotated between crossed polarizers. When it comes to the quality of the polymerized structure, some minor imperfections appear at the borders between different stripes, which are thought to be either a manufacturing defect in the azo dye layer or, less likely, a result of interaction between the polymer and azo dye.

It is worth emphasizing that the presented two-step process was completely automated by our own software. It means that much more complicated patterns can be fabricated. Here, we decided to combine four different azimuths with four tilt angles only for clarity in the presentation. However, we can define a much higher number of steps for both photoalignment and photopolymerization, but of course, the whole process would be more time-consuming. In such a way, we can even create a structure in which each pixel could have a unique combination of azimuth and tilt. On the other hand, the proposed method can be adopted in mass production for fast replication of some simple components (i.e., by using phase masks or micro-polarizer masks in the photoalignment step and by using patterned three-dimensional electrodes generating a non-uniform electric field in the photopolymerization step).

## 4. Discussion and Conclusions

The presented research demonstrates an experimental method for obtaining full 3D control of nematic LC’s ordering through the combination of patterned photoalignment and selective polymer stabilization to independently control both the azimuth and tilt of LC molecules. To our knowledge, it is the first practical demonstration of a truly three-dimensional patterned alignment of nematic LCs. Other types of liquid crystals, like cholesterics or blue phases, have a ‘natural’ ability to create three-dimensional local changes of the director; however, it mainly results from intrinsic properties of the LC, thus usually resulting in some periodicity or defects. With such materials, it is practically impossible to arbitrarily control the three-dimensional ordering of molecules, not to mention the lack of possibility of creating patterned alignment with micron-sized pixels. Our approach allows for the fabrication of structures with precise local control of tilt and azimuth of the molecules with high resolution, as our setup is based on a high-resolution DMD chip. Of course, this technique could be improved by using DLP-based illumination with even higher resolution or other techniques of patterned illumination (i.e., interferometric, holographic, based on SLMs, etc.).

It is worth emphasizing that although the polymer-stabilization process is used, it is still possible to retune the tilt of the molecules. It simply requires higher tuning voltages, or more specifically, higher intensity of the electric field, than in the case of non-polymerized areas of the sample. This property also opens up the possibility of creating new devices with a non-uniform response to the steering voltage, where some areas would be tuned in a ‘low-voltage’ regime, whereas others could be activated in a ‘higher-voltage’ mode. On top of that, the polymer network gives broader temperature stability of the nematic phase, which expands the application possibilities.

We believe that this research can form a foundation for fabricating complex LC-based optical elements and photonic devices with improved performance. In the next step, we will use the experimental techniques presented in this paper to fabricate new types of tunable lenses. We believe that three-dimensional control of the LC molecules’ alignment should allow us to obtain not only ‘standard’ converging and diverging lenses (including cylindrical ones) but also more complicated ones like tunable bifocal, trifocal or progressive lenses (as an active element of a new generation of smart corrective glasses). Moreover, it seems that other tunable optical components could be effectively and relatively easily fabricated, including tunable axicons, prisms, gratings, and waveplates. Finally, we believe that it will even be possible to create high-quality tunable holograms.

## Data Availability

The original contributions presented in this study are included in the article. Further inquiries can be directed to the corresponding authors.
